# A Simple, Low-Cost Platform for Real-Time Isothermal Nucleic Acid Amplification

**DOI:** 10.3390/s150923418

**Published:** 2015-09-16

**Authors:** Pascal Craw, Ruth E. Mackay, Angel Naveenathayalan, Chris Hudson, Manoharanehru Branavan, S. Tariq Sadiq, Wamadeva Balachandran

**Affiliations:** 1 College of Engineering, Design and Physical Sciences, Brunel University London, Kingston Lane, Uxbridge UB8 3PH, UK; E-Mails: eesrrem@brunel.ac.uk (R.E.M.); angel.naveenathayalan@brunel.ac.uk (A.N.); chris.hudson@brunel.ac.uk (C.H.); Manoharanehru.Branavan@brunel.ac.uk (M.B.); emstwwb@brunel.ac.uk (W.B.); 2 Oceans and Atmosphere flagship, Commonwealth Science and Industrial Research Organisation (CSIRO), Hobart, Tasmania 7001, Australia; 3 Institute for Infection and Immunity, St George’s University of London, Cranmer Terrace, London SW17 0RE, UK; E-Mail: ssadiq@sgul.ac.uk

**Keywords:** point-of care, molecular diagnostics, isothermal amplification, nucleic acid amplification test (NAAT), helicase dependent amplification

## Abstract

Advances in microfluidics and the introduction of isothermal nucleic acid amplification assays have resulted in a range of solutions for nucleic acid amplification tests suited for point of care and field use. However, miniaturisation of instrumentation for such assays has not seen such rapid advances and fluorescence based assays still depend on complex, bulky and expensive optics such as fluorescence microscopes, photomultiplier tubes and sensitive lens assemblies. In this work we demonstrate a robust, low cost platform for isothermal nucleic acid amplification on a microfluidic device. Using easily obtainable materials and commercial off-the-shelf components, we show real time fluorescence detection using a low cost photodiode and operational amplifier without need for lenses. Temperature regulation on the device is achieved using a heater fabricated with standard printed circuit board fabrication methods. These facile construction methods allow fabrications at a cost compatible with widespread deployment to resource poor settings.

## 1. Introduction

For many infectious diseases, timely, accurate and rapid diagnosis coupled with prompt effective treatment may improve patient outcomes and reduce transmission through vulnerable populations. Nucleic acid amplification tests (NAATs) offer sensitive and specific diagnosis often within 2 h, a fraction of the time taken for previous culture based methods. More recently, NAATs have been increasingly translated from requiring trained personnel and complex, expensive laboratory infrastructure to truly integrated sample-to-answer diagnostics, which can be used at the point-of-care. However, devices currently in use for these applications, such as the Cepheid GeneExpert [1] are too expensive for widespread deployment in resource poor settings and lack hand-held portability. Novel microfluidic technology, integrated with microelectronics and novel biotechnology will be the driving force, which will enable the benefits of NAAT to be realised in portable devices. In order to prove clinically beneficial, the performance of such test must equal or exceed existing strategies. The development and manufacture of accurate, portable and low cost, rapid NAAT testing devices requires novel and economical approaches.

Polymerase chain reaction (PCR), first described in 1985 [2], remains the most widely used method for nucleic acid amplification. More recently a range of isothermal nucleic acid amplification strategies have been developed to perform NAAT assays at a single temperature, a thorough review has been published by the group previously [3]. These isothermal NAAT techniques employ biochemical methods to mediate DNA helix strand separation and primer annealing that would otherwise require complicated, power-hungry thermal cycling hardware. The isothermal NAAT format is ideally suited for simple, low-cost portable devices.

Many strategies have been employed to detect products of nucleic acid amplification in NAATs and provide a result to the operator; Fluorescence [4], turbidity [5], capillary and gel electrophoresis [6], lateral flow [7], chemi-luminescent and electrochemical [8]. Of these methods, fluorescence detection is most widely employed [9] in point of care technology and one of few methods to be able to provide analyte quantification. Most NAATs require lenses to direct light onto a photodiode and increase weak fluorescence signal from amplified nucleic acid.

A fully automated, sample-to-answer device is being developed for detection of sexually transmitted infections (STIs) by the DoCLab group at Brunel University. The work, presented here, uses a plastic optical fibre to direct light onto the photo diode, thus reducing system complexity and cost [10,11]. Myers *et al.* showed a low cost handheld point of care device which does not require lenses [10]. The device uses an indium tin oxide plate as a heater which this takes 20 min to reach temperature however the platform presented within this paper uses a low cost printed circuit board (PCB) for heating which takes <120 s to reach temperature [10]. Sample loading within Myers device takes around 1 h whilst within the current platform sample loading is completed in <2 min [10]. Jenkins *et al.* use machined aluminium to act as heating blocks in an isothermal amplification platform. Whilst the system is low cost ($200) the use of PCR tubes limits the integration of sample preparation which is currently being developed in the next iteration of the proposed device [11]. The LabTube and LabReader system automates sample preparation using a centrifugal method within a modified 50 mL centrifuge tube [12]. The purified DNA is automatically transferred to a PCR tube. This tube is manually transferred to the LabReader which can run two isothermal or PCR reactions simultaneously [12]. This is fewer than the presented platform and excitation filters are required between the LEDs and the PCR tube [12]. The cost of the LabReader is expected to be one order of magnitude lower than the GeneXpert which is significantly more expensive than the presented low-cost platform [12,13]. Jiang *et al.* utilized solar heating to enable PCR in resource limited settings with a smartphone reader [14]. The main inhibiting factor is the requirement of a large lens and tilting platform to focus sunlight onto the microfluidic chip which will be impractical in many settings. It does however show that a smartphone can be used for both control and data acquisition [14]. This paper shows a low-cost module which performs real time isothermal NAATs on a platform that is easy to fabricate, using commercial off-the-shelf (COTS) components, and can be incorporated into a future integrated device at a cost that is compatible with widespread deployment in both wealthy and resource poor settings.

## 2. Experimental Section

### 2.1. Design of Microfluidic Chip

**Figure 1:**
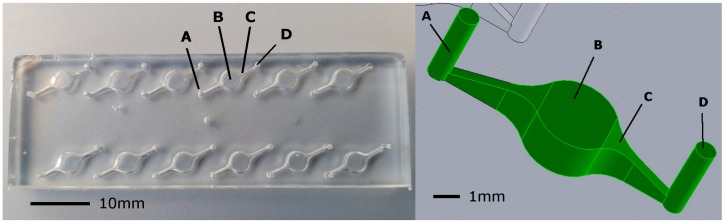
The prototype microfluidic chip. (**Left**) a photo (plan view) of the prototype chip showing (A) 1 mm diameter inlet port into which reaction mix was pipetted; (B) 25 µL reaction chamber: 4 mm diameter × 2 mm depth; (C) chamfered channel to prevent bubble trapping and (D) 1 mm outlet port; (**Right**) an isometric view of microfluidic reaction chamber (coloured green) to illustrate the chamfered channel geometry.

NAAT reactions were carried out on a glass and polydimethylsiloxane (PDMS) microfluidic device housing six planar circular chambers. Each reaction chamber was 4 mm diameter and 2 mm deep with a total volume of 25 µL. The six reaction chambers were spaced 11 mm apart and centered 4.5 mm from the chip edge to allow LED excitation from the chip side. Two 1 mm diameter inlet/outlet ports were connected to the chamber via chamfered channels (Figure 1) through which reaction mix was pipetted using standard pipette tips. The chamfered channel was necessary to prevent bubble trapping in reaction chambers. The microfluidic devices used for evaluations were reversible and could be used twice for two runs of six reactions each. To prevent carry over contamination, reaction chambers were not reused. It is envisaged that a final device would incorporate single use injection molded, polymer microfluidic cartridges which can be mass produced at a low cost. The current design can be readily transferred to mass manufacturing methods. A Solidworks CAD model (Dassault Systemes, Velizy, France) was fabricated using a 3D printer (Objet30 Pro, Stratasys, Eden Prairie, MN, USA) to generate a mold which was used to form PDMS (QSil218, ACC Silicones, Bridgewater, UK) casts. For these casts, 12.5 g PDMS was mixed at 1:10 (QSil 218A:QSil 218B) and placed in a centrifuge for 3 min at 2000 g to remove air bubbles. Cast PDMS was subsequently bonded to 75 mm × 25 mm × 1 mm glass microscope slides using a corona treatment method described elsewhere [15]. The cast PDMS was placed in an oven to cure for 3 h at 50 °C. Final chip thickness was 5 mm (1 mm glass slide plus 4 mm PDMS).

**Figure 2:**
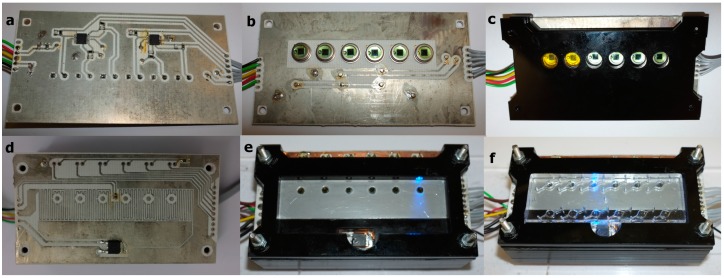
Layered structure of the device. (**a**) Underside of photodiode printed circuit board (PCB) layer containing filtered op-amp circuit; (**b**) Upper side of photodiode PCB layer where the six photodiodes are mounted, surrounded by a large ground plane; (**c**) Lower PMMA layer holding long pass emission filter in place over the photodiode; (**d**) SCOB heating element layer showing serpentine element and the six locations where the optical fibre passes through board (not drilled in picture); (**e**) Assembled stack of layers showing isothermal plate in position above the SCOB serpentine, optical fibres passing through the isothermal plate and the chip surround layer in place; (**f**) Assembled device with chip in place.

### 2.2. SCOB Heating Element

To enable chip heating, we developed a 35 µm thick, 300 µm wide serpentine copper track heating element, termed a SCOB element (Serpentine, Conducting, On-Board Element) shown in Figure 2d. This was made using standard photoetching methods from an economy two-layer printed circuit board (PCB) (Fotoboard 2, Precision Micro, Birmingham, UK). In addition, a surface mount thermistor was positioned centrally on the PCB to monitor heater temperature change and provide a signal for feedback control. A 3 mm thick aluminium plate, sized to cover the lower surface of the microfluidic chip, was attached to the SCOB heater using heat-transferring adhesive (TermoGlue, Termopasty Grzegorz Gasowski, Poland) as shown in Figure 2e. This served as an isothermal plate to distribute the heat generated from the SCOB heater evenly to the chip and thermistor. The thermistor and isothermal plate temperatures were calibrated and measured against a commercial instrument Testo 720 (Testo AG, Lenzkirch, Germany). Investigations into temperatures of individual chambers was conducted using a custom microfluidic chip with small thermistors embedded in the reaction chamber and read using a custom built six channel thermometer, calibrated against the Testo 720 instrument. Joule heating, due to current passing through the SCOB element, provided sufficient energy to reach 65 °C (the target temperature for isothermal amplification) in about 90 s. The MOSFET driving the SCOB element was mounted to the upper circuit board (see Figure 2) via a copper PCB layer that extended to the chip, allowing the heat generated in the MOSFET to contribute to chip heating.

### 2.3. Fluorescence Detection

**Figure 3:**
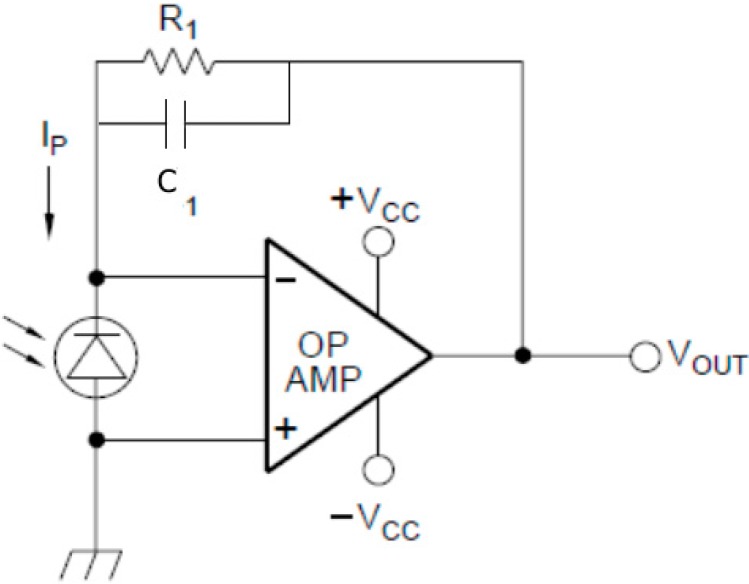
The photosensor circuit. A schematic of the low-cost photosensor circuit showing feedback resistor R_1_ and noise filtering capacitor C_1_.

In order to circumvent need for expensive amplified photodiodes, a simple circuit combining a low-cost (GBP 7.41) photodiode (BPW21, Centronic, Surrey, UK) with a high gain operational amplifier (OPA4750, Texas Instruments, Dallas, TX, USA) with 1G Ohm feedback resistor was employed, see Figure 3. The sensitivity of the photodiode system was evaluated and compared to the more expensive integrated amplified photodiodes SD-112-45-221 (GBP 82, Advanced Photonix, Ann Arbor, MI, USA) and OPA-6WB-500M (GBP 47, Optodiode Corp., Camarillo, CA, USA). This was done by placing the photosensors under known levels of irradiance produced by a diffused 525 nm LED at the emission wavelength of the DNA dye used (EvaGreen, Biotium, Hayward, CA, USA). Calibration was performed using a laser power meter (Nova, Ophir Photonics, North Logan, UT, USA) to determine the corresponding irradiance.

**Figure 4:**
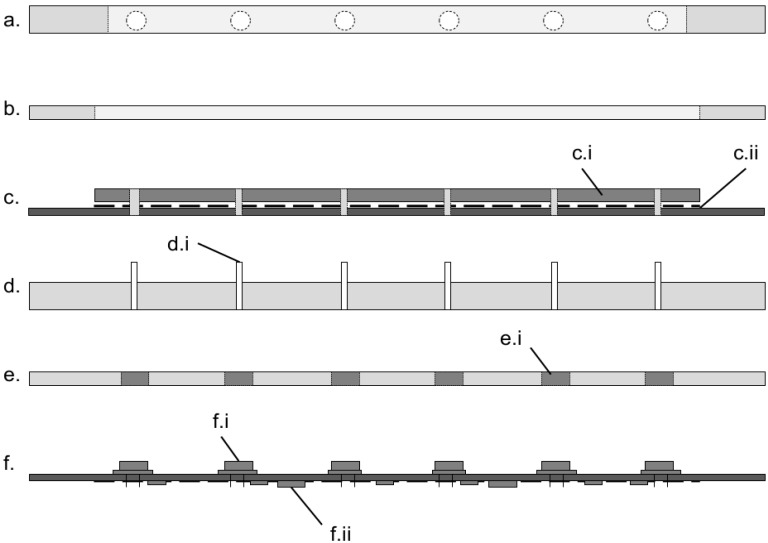
Schematic diagram showing the layers (exploded view) that form the device. (**a**) The 5 mm PMMA chip surround layer with LEDs shown in alignment holes, this layer holds the chip in place over the optical fibres (not shown) and aligns the LEDs with the reaction chambers; (**b**) The 3 mm PMMA layer that surrounds the isothermal plate; (**c**) The upper PCB layer with isothermal plate (c.i) glued in position above the SCOB element (dashed line, c.ii) and the PCB; (**d**) Optical fibre layer (PMMA) with 3000 μm optical fibres (d.i) in place; (**e**) The filter PMMA layer which holds the long pass emission filter (e.i) in place over the photodiodes; (**f**) The lower PCB with photodiodes mounted on the upper surface (f.i) and the amplifcation circuitry on the lower surface (f.ii).

Within the device, the photodiode was coupled to the chip via a 15 mm section of hand polished, unjacketed 3000 µm plastic optical fiber (Edmund Optics, Barrington, NJ, USA) illustrated in Figure 4d.i. These low-cost plastic optical fibres were used in place of lenses to avoid dead air spaces where dust could accumulate, to increase resilience of the device to shock encountered during field use and to simplify the manufacturing process. Between the photodiode and the optical fibre was a 8 mm diameter long pass filter cut from low-cost orange glass (OG515, Schott AG, Mainz, Germany) shown in Figure 4e.i, this had a wavelength cutoff λ_c_ = 515 nm. The optical fibre passed from the photodiode/optical filter, through a hole on the SCOB heater board and underside of the isothermal plate to rest flush with the upper surface of the isothermal plate where the underside of the microfluidic chip chambers were located (Figure 2e). Excitation was performed at 470 nm using a 3 mm LED (L-7104QBC-D, Kingbright, New Taipei City, Taiwan). These were mounted on the upper circuit board and illuminated the reaction chamber from the side of long axis of the chip, orthogonal to the optical fibre. It was found that this orthogonal excitation resulted in less crosstalk from the excitation LED into the sensor photodiode. Both illumination from the lower surface (45° from photodiode plane) and upper surface (135° from photodiode plane) of the chip showed a reduction in the signal to blank ratio of the sensor from 1.8 to 1.25.

The use of optical fibres allowed the photodiodes to be located on a second PCB layer below the SCOB PCB layer, Figure 2f. A ground plane on the upper surface of the lower layer that acted to shield the very sensitive, high gain amplification circuitry on the underside of the photodiode PCB, Figure 4f.ii, from electromagnetic interference (EMI) generated from the SCOB element, which was identified as a considerable noise component during early designs.

### 2.4. Control and Signal Acquisition

The control of the SCOB heating system and excitation LEDs, in addition to the acquisition and processing of sensor output, was performed using an Arduino Mega2560 microcontroller board (Arduino, Turin, Italy). Software written in the Arduino environment was used to initiate excitation LEDs and acquire 20 photosensor readings over 300 ms and return an average of readings following a pre-sampling delay to allow for sensor rise time. Following sampling, an 800 ms delay allows the sensor to return to baseline. Simple averaging of sensor signal is sufficient to mitigate effect of external noise and noise arising from intermittent current in SCOB heating circuitry. Fluorescence signal sampling from each chamber was performed in serial order to prevent crosstalk between different reaction chambers. Control of the SCOB heater was performed using a simple bang-bang control algorithm which triggered in response to thermistor temperature on the SCOB element. For analysis, data output from the microcontroller, time stamp (seconds) and raw fluorescence signal (0–5000 mV), were recorded to study change in fluorescence of the reaction over time. A baseline fluorescence for each reaction was determined as mean signal over the first 10 min of data acquisition. Baseline for each reaction was then subtracted from raw data to give change in fluorescence for each sample. Acquired real-time data was displayed on a PC for analysis. To reduce cost, future devices could provide a binary, positive/negative output indicator or connect to PC/wireless network for further analysis.

### 2.5. Helicase Dependent Amplifcation (HDA)

A one step thermophillic HDA (tHDAIII Universal tHDA kit, BioHeix, MA, USA) was performed on chip using reagent ratios described in the manual [16] to prepare a 25 µL reaction. The reaction was assembled in a 200 µL microcentrifuge tube, pipetted into the microfluidic chip by hand and incubated at a target temperature of 65 °C. For purposes of platform evaluation, primers and positive control template supplied with the tHDAIII kit were used. Primers, NGF3 (forward) and NGR3 (reverse), were used at a final reaction concentration of 7.5 nM each and 1 ng control template pCNG1 was added. Once reaction mix was in the microfluidic chip, the chip was sealed using a silicone elastomer (Blu-stuff, UK) which had been cast in a 3D printed mold. The sealed chip was placed on the preheated isothermal plate of the detection device and data collection algorithm initiated. If inlets and outlets were not sealed correctly bubble formation occurred within the chamber, greatly impairing optical results and forcing assay reaction volume out of the chamber. To ensure test specificity, electrophoresis of reaction mix at endpoint was run on 2% agarose gels with ethidium bromide stain.

### 2.6. Platform Construction

The device described herein was assembled from six layers of laser cut 5 mm and 3 mm PMMA (Perspex) as described in Figure 2. This fabrication method used rapid prototyping and building, on low budgets without access to manufacturing infrastructure, and could be easily replaced with one or two injection moulded parts for mass production, the cost of components used in the system is outlined in Table 1. As outlined in Figure 2, the upper two PMMA layers were placed above the upper circuit board, which had the SCOB element and isothermal plate. These layers formed a recess into which the microfluidic chip could be placed. Insertion of the chip into the recess aligned the chip with excitation LEDs and detection optical fibres without need for user intervention. The microfluidic cartridge can be placed accurately and repeatedly into this recess by hand, due to the large optical fibre aperture; no loss in optical signal is seen. The LEDs were mounted horizontally in the top layer, orthogonal to the six 3000 µm plastic optical fibres which ran through the layers between the upper and lower PCBs. These optical fibres coupled the underside of the reaction chambers in the microfluidic chip to the bandpass filter and photodiode assembly mounted on the top side of the lower PCB.

**Table 1:** Bill of materials for the device and microfluidic cartridges. A large portion of the cost is in the Arduino microcontroller board used for control and data acquisition during the research process, in a production device this would be replaced with a single microcontroller chip as part of the circuitry at a much lower cost.

	Item	Cost (GBP)
**Hardware**	PMMA	4.00
	Isothermal plate	1.30
	Fasteners	0.40
**Electronics**	Operational Amplifier	4.60
	MOSFET	0.49
	Passive components	7.00
	LEDs	3.75
	Photodiodes	44.46
	Arduino Mega2560	35.58
	PCB	2.28
**Optical Components**	Filter	1.63
	Optical fibre	0.73
**Microfluidics**	Microfluidic mold	27.38
	Microscope slide	0.14
	PDMS	0.93
**Consumables**	PCR tube/pipette tips	0.85
	**Total**	**135.52**

## 3. Results and Discussion

### 3.1. SCOB Heating Element Stability

**Figure 5:**
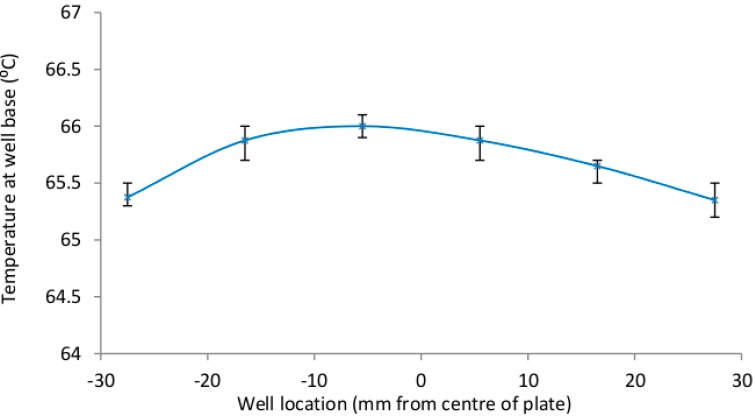
The temperature of the six reaction chambers is shown with error bars indicating the maximum and minimum values reached over the 5 repeat measurements. The maximum and minimum temperatures were 65.2 °C and 65.7 °C. All of the measurements were within the 60–70 °C range (shown here as the y axis range) which we had shown successful tube-based HDA amplification.

**Figure 6:**
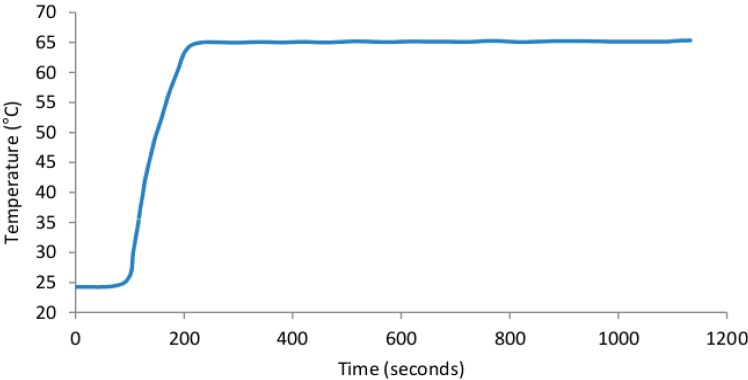
Isothermal plate heating and temperature stability characteristics. The device was heated from room temperature to operating temperature (65 °C) with continual temperature measurements to characterise the thermal properties over long time spans.

**Figure 7:**
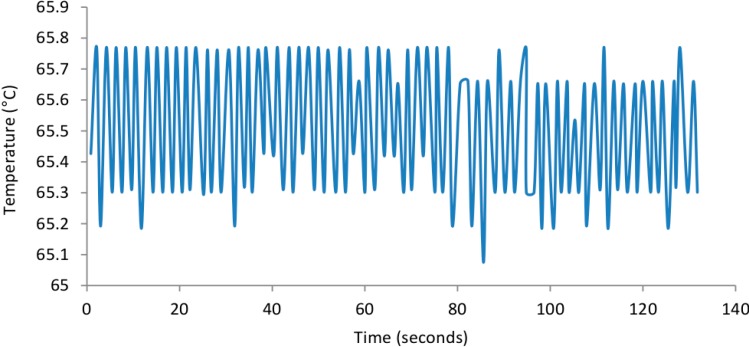
Isothermal plate temperature in high resolution over ~2 min. The device was heated to operating temperature and high resolution temperature measurements were taken from within a filled well to identify the thermal variation over short time periods.

**Figure 8:**
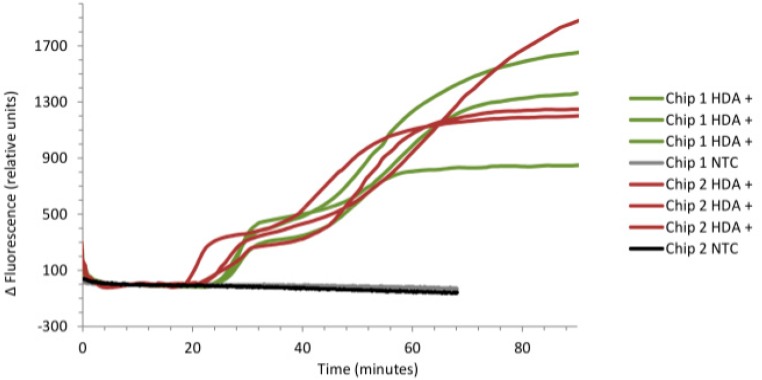
qHDA amplification curves, showing clear differentiation between positive and negative reactions for 1 ng pCNG1.

The SCOB heating element described provided stable heating, within the desired 65–67 °C range, of all six microfluidic chip reaction chambers (shown in Figure 5, Figure 6 and Figure 7). As predicted there was a very small 0.8 °C gradient from the central portion of the isothermal plate to lateral portions (Figure 5) believed to be due to conductive heat loss at plate edges. Figure 6 shows rapid rise to operating temperature of ~120 s and subsequent temperature stability over 15 min of operation. Figure 7 shows a higher resolution view of temperature oscillations once operating temperature has been reached. Accurate amplification of all positive samples (Figure 8) suggests that this temperature difference is of no consequence for binary result, positive/negative type detections described here. For quantitative use, this gradient may adversely impact accuracy. Power consumption of the SCOB element was 24 W when heating, the duty cycle was slightly less than 0.5 resulting in a mean consumption over reaction time of ~12 W. Power consumption will be further reduced in subsequent iterations by reducing large chip and isothermal plate sizes used here with more minimal geometries.

### 3.2. Fluorescence Detection

**Figure 9:**
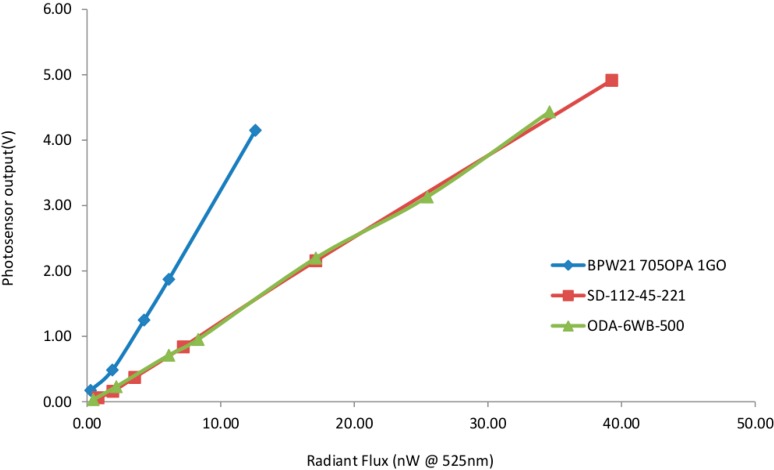
Sensitivity of low-cost photosensor system used in this research in comparison to existing integrated photodiode/amplifiers systems used elsewhere. The greater sensitivity of the low cost photosensor, BPW21 705OPA 1GO (blue diamonds) can be seen in the greater photosensor output signal per radiant flux as compared to the commercial photodiodes SD-112-45-221 and ODA-6WB-500.

The low-cost photosensor system was shown to have higher sensitivity than that of more expensive amplified photodiodes (Figure 9) used elsewhere [17]. However, this photosensor system did have a much longer rise time of 800 ms compared with the expensive integrated devices which both peaked within 15 ms. To ensure readings were not taken during photosensor circuit rise time, a 800 ms delay from the time excitation LEDs were triggered to when sensor reading was taken was included. This longer duration of excitation did not lead to detectable photo-bleaching of intercalating dye used at 30 s sampling intervals for 90 min.

### 3.3. Real-Time Detection of pCNG1

The system described detected 1 ng of pCNG1 template in all positive samples in 20–30 min. There was high level of repeatability across different chambers (Figure 8) and microfluidic chips. Slight differences in hand polished optical fibres may have led to different optical coupling efficiency between chip, fibre and filter and would explain inter-sensor variance seen in final fluorescence level. Experiments were run in parallel using the commercially available Axxin T16-ISO platform which showed comparable reaction kinetics. By using off the shelf fibres in future versions of the device this discrepancy would be eliminated which will be particularly important when analyzing real clinical samples.

As absolute fluorescence of dye is measured, rather than a signal normalised against a reference dye (ΔRn), there is possibility that temperature variation may play a role. However, as none of the variations seen correlated with temperature variations measured it seems more likely that inter-sensor variation result from optical coupling differences, which is unsurprising given the simplicity of our optical systems and hence its low cost. As there is large difference (800–1700 mV) between all positives and negatives, inter-positive differences are of little significance in presence/absence testing.

Gel electrophoresis at end point showed that target had amplified in the positive control with no evidence of primer artefacts with blank negatives, corroborating the results seen in fluorescence measurements. The one-off cost of this chip-based system is about US$60. This is a significant cost difference to the most advanced existing single test NAAT technologies such as the Cepheid Omni [13]. With further refinements, including the integration of a microcontroller into the circuitry to remove the need for the relatively expensive Arduino development boards, it is possible to manufacture a system for less than $40, further saving would be possible with volume manufacturing.

## 4. Conclusions

The ability to perform rapid field based NAATs has potential to transform clinical and public health medicine. For the benefits of such technology to be implemented, these must come at reasonable cost. This is particularly pertinent for use in the developing world. This paper describes the use of low-cost, COTS components and manufacturing techniques which can be applied by a modestly equipped institution to build a low-cost device for isothermal NAAT testing. Whilst this is not a complete diagnostic solution it serves as an enabling technology to bring such devices to market at a lower cost. The simple, low-cost SCOB heating element demonstrated temperature control within desired temperature limits with low power consumption on a platform that is easy to fabricate. The simple optical system was demonstrated to provide high sensitivity detection of the qHDA reaction and the performance of the device was evaluated and shown to provide rapid, reproducible and unequivocal results when evaluated with the pCNG1 control reaction. The system has been tested with discrete samples loaded manually onto the chip, automation of the device is on-going; results are shown for one concentration of DNA, further experiments must be conducted at lower DNA concentration. The system shown represents one module of a complete sample-in to answer-out system in development. Sample preparation will occur within the microfluidic device, passive mixing will be conducted within microfluidic channels and nucleic acid extraction is done on a cationic membrane [18]. Active device cooling is not required for repeated runs, including when sample preparation is integrated, as any additional temperature will aid cell lysis and will not inhibit nucleic acid purification. We believe that this advance in low-cost, low-tech instrumentation will allow the benefits of high tech diagnostics to be taken into the field at a price compatible with widespread use.
